# PGN and LTA from *Staphylococcus aureus* Induced Inflammation and Decreased Lactation through Regulating DNA Methylation and Histone H3 Acetylation in Bovine Mammary Epithelial Cells

**DOI:** 10.3390/toxins12040238

**Published:** 2020-04-09

**Authors:** Yongjiang Wu, Jingbo Chen, Yawang Sun, Xianwen Dong, Zili Wang, Juncai Chen, Guozhong Dong

**Affiliations:** 1 College of Animal Science and Technology, Southwest University, Beibei District, Chongqing 400716, China; wuyongjiang@email.swu.edu.cn (Y.W.); c10417511@email.swu.edu.cn (J.C.); syaw507@swu.edu.cn (Y.S.); wzl9698@swu.edu.cn (Z.W.); juncaichen@swu.edu.cn (J.C.); 2 Institute for Herbivorous Livestock Research, Chongqing Academy of Animal Science, Chongqing 402460, China; chenghuafen@email.swu.edu.cn

**Keywords:** peptidoglycan, lipoteichoic acid, DNA methylation, histone acetylation, inflammation, lactation, bovine mammary epithelial cells

## Abstract

*Staphylococcus aureus* (*S. aureus*) and *Escherichia coli* (*E. coli*) are the most common pathogens of mastitis, and *S. aureus* generally causes subclinical mastitis which is more persistent and resistant to treatment. Peptidoglycan (PGN) and lipoteichoic acid (LTA) are cell wall components of *S. aureus*. Although the roles of PGN and LTA in causing inflammation are well studied, the epigenetic mechanisms of the effects of PGN and LTA on the inflammation and lactation remain poorly understood. This study characterized the gene expression profiling by RNA sequencing and investigated DNA methylation and histone acetylation in relation to inflammation and lactation in the immortalized bovine mammary epithelial cell line (MAC-T). The cells were cultured for 24 h with neither PGN nor LTA (CON), PGN (30 μg/mL), LTA (30 μg/mL), and PGN (30 μg/mL) + LTA (30 μg/mL), respectively. The number of differentially expressed genes (DEGs) and the expression of proinflammatory factors including interleukin (IL)-1β, IL-6, IL-8, chemokine (C-X-C motif) ligand (CXCL)1, and CXCL6 of the treatments increased in the following order: CON < PGN < LTA < PGN + LTA, and the DEGs mainly enriched on the cytokine-cytokine receptor interaction and chemokine signaling pathway. LTA and PGN + LTA induced hypomethylation of global DNA by suppressing DNA methyltransferase (DNMT) activity. PGN and LTA, alone or combined, decreased the mRNA expression of casein genes (*CSN1S1, CSN2,* and *CSN3*) and the expression of two caseins (CSN2 and CSN3), and reduced histone H3 acetylation by suppressing histone acetyltransferase (HAT) activity and promoting histone deacetylase (HDAC) activity. Collectively, this study revealed that PGN and LTA induced inflammation probably due to decreasing DNA methylation through regulating DNMT activity, and decreased lactation possibly through reducing histone H3 acetylation by regulating HAT and HDAC activity in bovine mammary epithelial cells.

## 1. Introduction

Mastitis is one of the most common and highly prevalent inflammatory diseases in dairy cows and it greatly increases economic losses by reducing milk yield and quality and increasing the cull rate of cows. For example, the economic losses associated with mastitis exceed 1.55 billion euros per year in Europe [1]. *Staphylococcus aureus* (*S. aureus*) and *Escherichia coli* (*E. coli*) are the most common pathogens of mastitis, and *S. aureus* generally causes subclinical mastitis which is more persistent and resistant to treatment. Peptidoglycan (PGN) and lipoteichoic acid (LTA) are cell wall components of *S. aureus.* The weak and persistent proinflammatory activity of LTA and PGN mainly causes subclinical and chronic mastitis [2,3,4] which is difficult to detect and cure. Previous studies have reported the effects of LTA and LTA + PGN on gene expression profiling of bovine mammary epithelial cells, and mainly focused on the proinflammatory activity of PGN and LTA [5,6,7]. However, the epigenetic modification mechanisms of the inflammation induced by PGN and LTA remain poorly understood, and the direct effects of PGN and LTA, alone or combined, on lactation of bovine mammary epithelial cells and the inherent epigenetic mechanisms receive little attention.

The expression of proinflammatory factors could be regulated by epigenetic modification [8,9]. Epigenetic modification mechanisms mainly include DNA methylation, histone modification, and noncoding RNA regulation, all of which regulate gene expression without changing DNA sequence. DNA methylation usually involves gene silencing [10] that typically represses gene transcription and is catalyzed by DNA methyltransferase (DNMT). On the other hand, the higher acetylation degree of histones tends to activate transcription, and histone acetylation is mainly regulated by histone acetyltransferase (HAT) and histone deacetylase (HDAC). HAT catalyzes histone acetylation, while HDAC catalyzes histone deacetylation. *E. coli* and its cell wall component lipopolysaccharide (LPS) could cause hypomethylation at many inflammatory loci by suppressing DNMT expression, and then increase the proinflammatory factor expression of porcine mammary epithelial cells [11], human dental pulp cells and macrophages [12,13], bovine fibroblasts [14], and so on. Our previous study showed that LPS enhanced immune responses of bovine mammary epithelial cells by decreasing DNA methylation [15]. Moreover, our other study indicated that LPS reduced histone H3 acetylation through enhancing HDAC activity, and subsequently suppressed the expression of lactation-related genes, such as acetyl coenzyme-A carboxylase 1 (*ACACA*), fatty acid synthase (*FASN*), and ribosomal protein S6 kinase 1 (*S6K1*) [16]. Like *E. coli*, *S. aureus* could induce bovine subclinical mastitis by regulating DNA methylation [17,18,19]. *S. aureus* also could cause a reduction in milk yield of dairy cows [20] and influence histone acetylation [21,22]. Therefore, we speculated that PGN and LTA might induce inflammation and decrease lactation through regulating DNA methylation and histone acetylation in bovine mammary epithelial cells. Thus, the present work was aimed at investigating the effects of PGN and LTA, alone or combined, on transcriptome and the expression of genes in relation to inflammation and lactation, and to explore the epigenetic modifications caused by PGN and LTA. 

## 2. Results

### 2.1. Effects of PGN and LTA on Transcriptome

#### 2.1.1. Overview of RNA Sequencing Data

**Figure 1:**
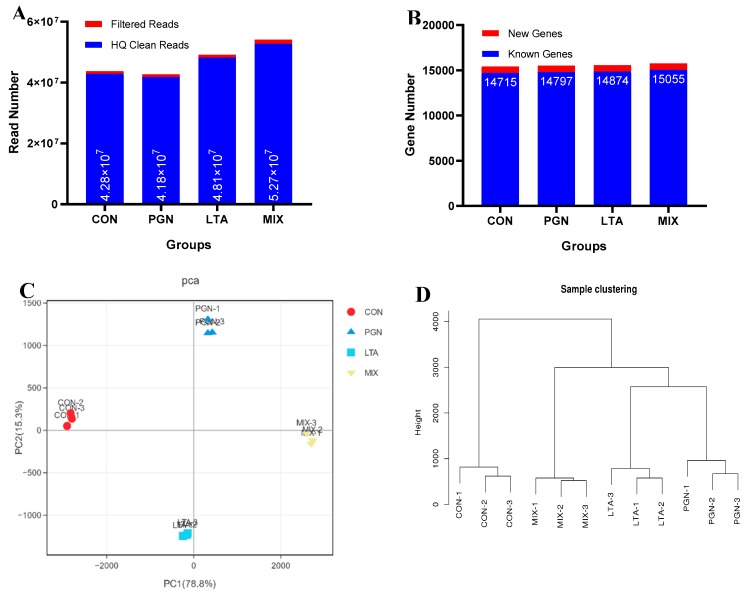
The analysis of RNA sequencing data. (**A**) The statistics of filtered and high-quality (HQ) clean read numbers. (**B**) The statistics of new and known gene numbers. (**C**) The principle component analysis (PCA) of gene expression profiles. (**D**) The sample clustering tree of gene expression profiles. CON, control group; PGN, peptidoglycan group; LTA, lipoteichoic acid group; MIX, PGN + LTA group.

RNA sequencing (RNA-Seq) produced a total of 4–5 million reads per sample, and the high-quality (HQ) clean reads represented more than 98% of all reads (Figure 1A). The quality of raw sequencing data was analyzed by FastQC (Table S1). The HQ clean reads were mapped against bovine reference genome (Bos Taurus, assembly ARS-UCD1.2), and the mapping ratio was more than 94%. A total of 14–15 thousand known genes and 6–7 hundred new genes were identified, and the known genes accounted for more than 95% of all identified genes (Figure 1B). The HQ clean reads of different treatments (or groups) increased in the following order: PGN < LTA < PGN + LTA, and the number of the known genes of different groups also increased in the above order. The gene expression profiles of each group are shown in Figure 1C,D. The principle component analysis (PCA) for gene expression profiles showed that the positions of three replicates in each group were very close to each other, whereas the four groups were clearly separated. This indicated little differences within each group, but great differences among the groups. The sample clustering analyses of gene expression profiles confirmed the PCA results. 

**Figure 2:**
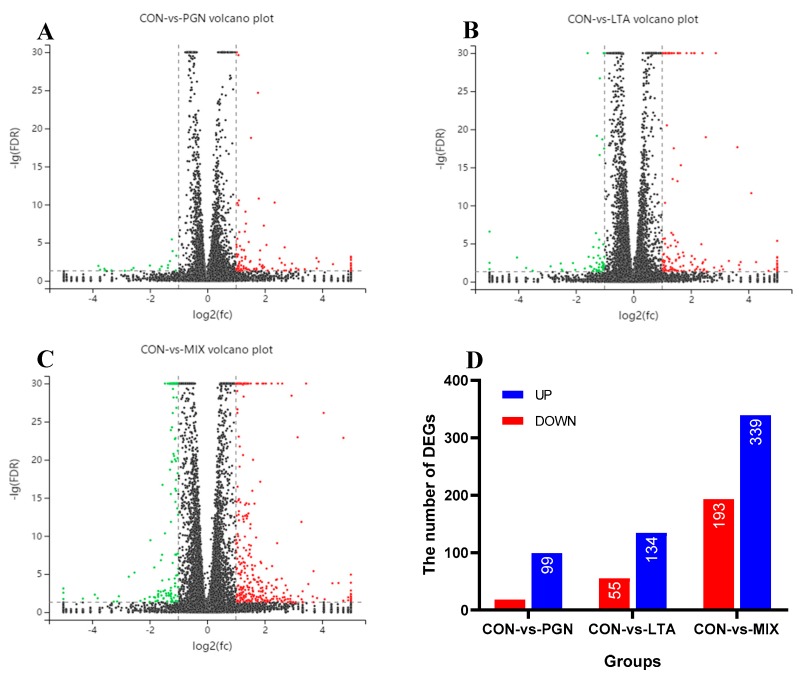
The statistics of differentially expressed genes (DEGs). (**A**) The DEGs Volcano plot diagram of the CON vs. PGN. The red dots, green dots, and black dots represent upregulated DEGs, downregulated DEGs, and non-significantly different genes, respectively. (**B**) The DEGs Volcano plot diagram of the CON vs. LTA. (**C**) The DEGs Volcano plot diagram of the CON vs. PGN + LTA. (**D**) The number of up- and down-regulated DEGs in the three conditions. CON, control group; PGN, peptidoglycan group; LTA, lipoteichoic acid group; MIX, PGN + LTA group; UP, upregulated DEGs; DOWN, downregulated DEGs.

The differences of gene expression between groups were analyzed by Edge R software (Release 3.10, http://www.bioconductor.org/packages/release/bioc/html/edgeR.html). The false discovery rate (FDR) and log_2_ fold change (log_2_ FC) were used to screen the differentially expressed genes (DEGs), and the screening conditions were FDR < 0.05 and |log_2_ FC| > 1. The red dots and green dots represent the up- and down-regulated DEGs, respectively, in the three volcano plots (Figure 2). As shown in the three volcano plots in the Figure 2A–C, there were significantly more red dots than green plots, indicating the number of upregulated DEGs was more than that of downregulated DEGs, compared with the CON group. The number of DEGs is shown in Figure 2D. Compared with the CON group, the number of up- and down-regulated DEGs of different treatments increased in the following order: PGN < LTA < PGN + LTA. The annotations of DEGs in the CON vs. PGN, CON vs. LTA, and CON vs. PGN + LTA conditions are shown in Tables S2–S4. 

#### 2.1.2. GO Function Annotation and KEGG Pathway Enrichment Analyses of DEGs 

**Figure 3:**
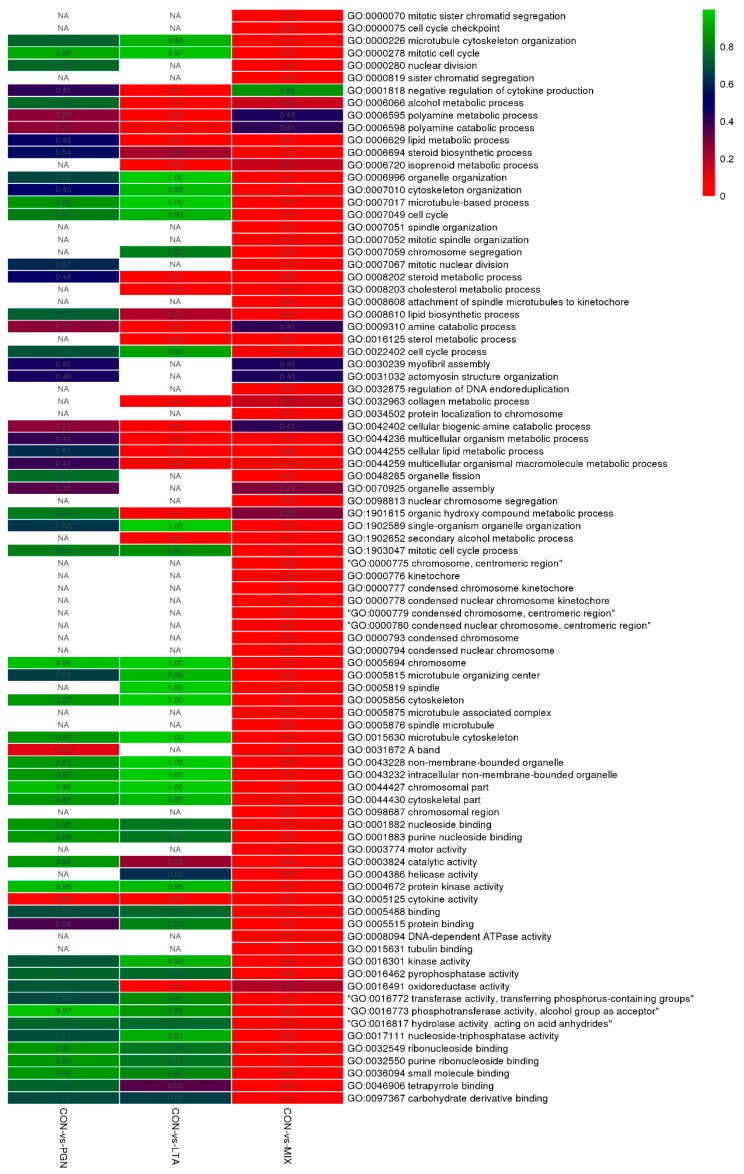
The heatmap of gene ontology (GO) enrichment for the DEGs in CON vs. PGN, CON vs. LTA, and CON vs. PGN + LTA conditions. Each column represents a comparison condition, and each row represents a GO enrichment term. The color gradients of green-red represent the significant levels from low to high. The number in the square lattice represents specific *p* values for the enrichment in the GO terms. NA indicates that no DEGs in the comparison condition enriched in the GO terms.

The biological function classifications for the DEGs were performed by gene ontology (GO) enrichment analyses. As shown in Figure 3, the DEGs in the CON vs. LTA and CON vs. PGN + LTA conditions significantly enriched (*p* < 0.05) in 19 and 75 GO terms, respectively. Although the DEGs in the CON vs. PGN condition did not significantly enrich in any GO term, they had a tendency (*p* = 0.057) of enriching in cytokine activity. The DEGs in the CON vs. LTA and CON vs. PGN + LTA conditions mainly significantly enriched in the cytokine activity term. Many non-significantly enriched GO terms under single stimulation with PGN or LTA became significant if the cells were co-stimulated with PGN and LTA. The top 10 GO molecular function, biological process, and cellular component terms of the DEGs in the CON vs. PGN, CON vs. LTA, and CON vs. PGN + LTA conditions are supplied in Tables S5–S7, respectively.

**Figure 4:**
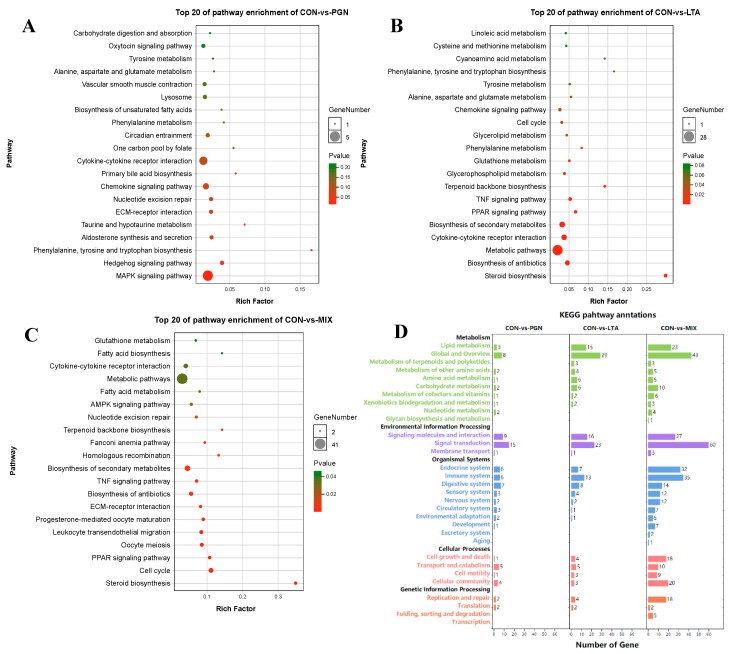
The bubble chart and histogram of Kyoto encyclopedia of genes and genomes (KEGG) pathway enrichment for DEGs. (**A**–**C**) The KEGG pathway enrichment bubble chart for the DEGs in the CON vs. PGN, CON vs. LTA, and CON vs. PGN + LTA conditions, respectively. (**D**) The number of the DEGs enriched in KEGG-B-class in the CON vs. PGN, CON vs. LTA, and CON vs. PGN + LTA conditions. CON, control group; PGN, peptidoglycan group; LTA, lipoteichoic acid group; MIX, PGN + LTA group.

Meanwhile, the major enrichment pathways of the DEGs were analyzed by Kyoto encyclopedia of genes and genomes (KEGG) pathway enrichment analyses. The top 20 pathways in the three conditions (CON vs. PGN, CON vs. LTA, and CON vs. PGN + LTA) are shown by the bubble charts in Figure 4A–C. The DEGs in the three conditions all mainly enriched in the cytokine-cytokine receptor interaction pathway (ko04060). The number of DEGs enriched in KEGG-B-class in the CON vs. LTA condition was more than that in the CON vs. PGN condition but was less than that in the CON vs. PGN + LTA condition (Figure 4D). The annotations of all KEGG pathways in the CON vs. PGN, CON vs. LTA, and CON vs. PGN + LTA conditions are supplied in Tables S8–S10, respectively. 

### 2.2. Validation of Selected DEGs by RT-qPCR Analyses

**Figure 5:**
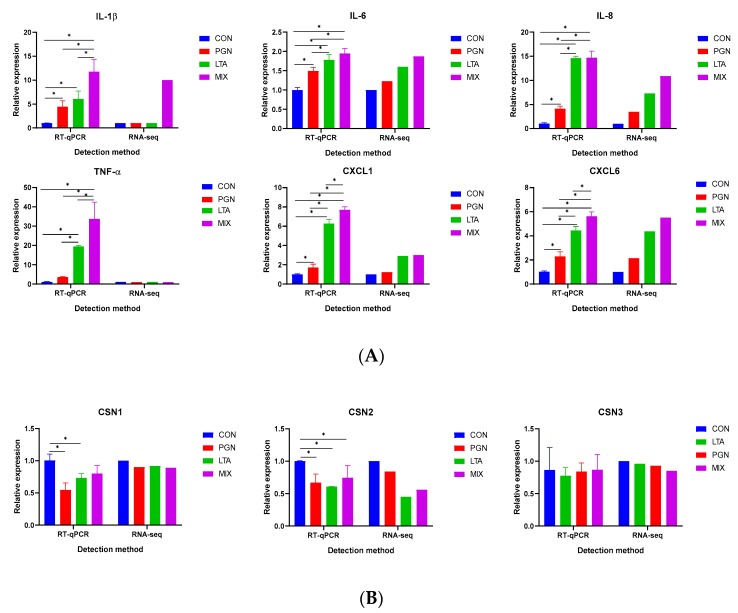
RT-qPCR validation of the differentially expressed genes (DEGs) obtained by RNA sequencing (RNA-Seq). (**A**) The genes involved in inflammation. (**B**) The genes involved in casein synthesis. Data represent the mean and standard deviation (*n* = 6), and the asterisk indicates statistical difference (* *p* < 0.05) between the indicated columns, based on one-way analysis of variance and Duncan’s range test. *IL-1β*, interleukin-1β; *IL-6*, interleukin-6; *IL-8*, interleukin-8; *TNF-α*, tumor necrosis factor-α; *CXCL1*, chemokine (C-X-C motif) ligand 1; *CXCL6*, chemokine (C-X-C motif) ligand 6; *CSN1S1*, αS1-casein; *CSN2*, β-casein; *CSN3*, κ-casein; RT-qPCR, reverse transcription quantitative real-time polymerase chain reaction; CON, control group; PGN, peptidoglycan group; LTA, lipoteichoic acid group; MIX, PGN + LTA group.

To validate the RNA-Seq data, a total of six inflammation-related cytokine and chemokine genes (interleukin (*IL*)*-1β*, *IL-6*, *IL-8*, tumor necrosis factor-α (*TNF-α*), chemokine (C-X-C motif) ligand (*CXCL*)*1*, and *CXCL6*) and three casein genes (αS1-casein (*CSN1S1*), β-casein (*CSN2*), and κ-casein (*CSN3*)) were selected for measuring the mRNA expression by reverse transcription quantitative real-time polymerase chain reaction (RT-qPCR) analyses. As shown in Figure 5, the relative mRNA expression of all these selected genes measured by RT-qPCR was basically consistent with the RNA-Seq data. The mRNA expression of proinflammatory genes of the groups increased in the following order: CON < PGN < LTA < PGN + LTA group (Figure 5A). As shown in Figure 5B, the mRNA expression of *CSN1S1* for the PGN and LTA groups significantly decreased, compared with the CON group. The mRNA expression of *CSN2* for the PGN, LTA, and PGN + LTA groups was significantly lower than that in the CON group. The mRNA expression of *CSN3* for the PGN, LTA, and PGN + LTA groups also was lower than that in the CON group, but the differences were not significant. 

### 2.3. LTA and PGN + LTA Reduced Global DNA Methylation Levels by Suppressing DNMT Activity

**Figure 6:**
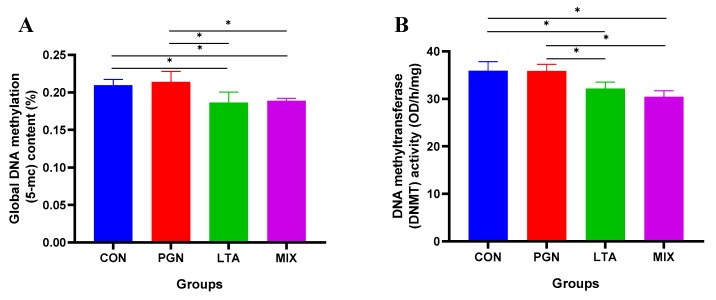
The global DNA methylation levels and methyltransferase (DNMT) activity. (**A**) The global DNA methylation levels. (**B**) The DNMT activity. The global DNA and nucleoprotein were isolated to measure the DNA methylation levels and DNMT activity by enzyme-linked immunosorbent assay (ELISA), respectively. Data represent the mean and standard deviation (*n* = 6), and the asterisk indicates statistical difference (* *p* < 0.05) between the indicated columns, based on one-way analysis of variance and Duncan’s range test. CON, control group; PGN, peptidoglycan group; LTA, lipoteichoic acid group; MIX, PGN + LTA group.

As shown in Figure 6, the global DNA methylation levels and DNMT activity in the LTA and PGN + LTA groups were significantly lower than those in the CON and PGN groups.

### 2.4. PGN and LTA Reduced Histone H3 Acetylation Levels by Suppressing HAT Activity

**Figure 7:**
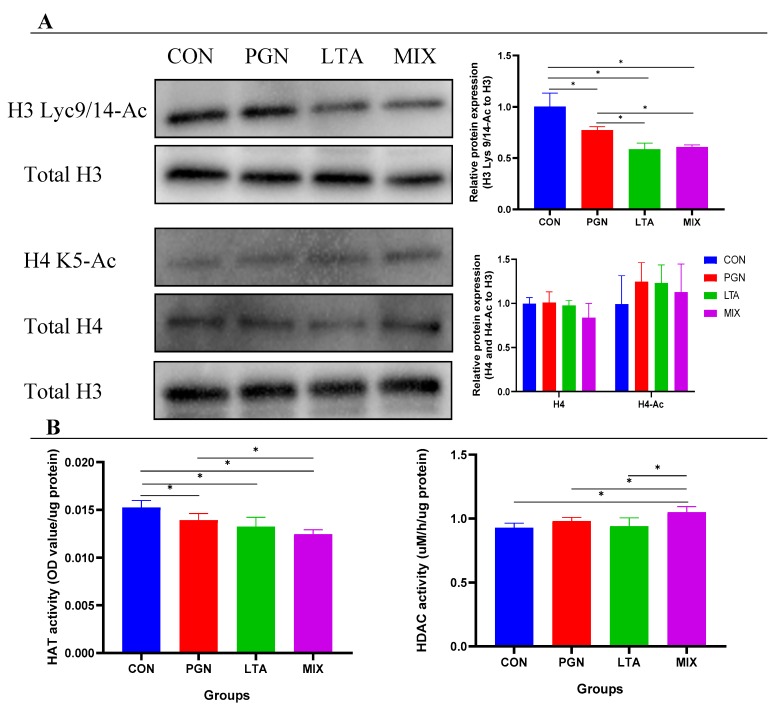
The histone (H3 and H4) acetylation levels and histone acetyltransferase (HAT) and histone deacetylase (HDAC) activity. (**A**) The protein expression of the histones (H3 and H4) and acetylated histones (H3 Lys9/14-Ac and H4 K5-Ac). (**B**) The activity of the HAT and HDAC. The nucleoprotein was isolated to examine histone acetylation levels and the activity of the HAT and HDAC using Western Blot analysis and enzyme-linked immunosorbent assay (ELISA), respectively. The histone H3 was selected as a housekeeping protein. Quantitation of blots is representative of three independent experiments. Data represent the mean and standard deviation (*n* = 6), and the asterisk indicates statistical difference (* *p* < 0.05) between the indicated columns, based on one-way analysis of variance and Duncan’s range test. H3, histone H3; H3 Lys9/14-Ac, histone H3 Lys9/14 acetylation; H4, histone H4; H4 K5-Ac, histone H4 K5 acetylation; CON, control group; PGN, peptidoglycan group; LTA, lipoteichoic acid group; MIX, PGN + LTA group.

As shown in Figure 7, the histone H3 Lys9/14 acetylation (H3 Lys9/14-Ac) levels in the PGN, LTA, and PGN + LTA groups were significantly lower than that in the CON group, but histone H4 K5 acetylation (H4 K5-Ac) levels were not significantly different among all the groups. Furthermore, the HAT activity of the PGN, LTA, and PGN + LTA groups significantly decreased, compared with the CON group. Moreover, the HDAC activity of the PGN + LTA group was significantly higher than that of the CON, PGN, and LTA groups.

### 2.5. PGN and LTA Promoted Inflammation But Reduced Lactation 

#### 2.5.1. PGN and LTA Increased Proinflammatory Factor Secretion 

**Figure 8:**
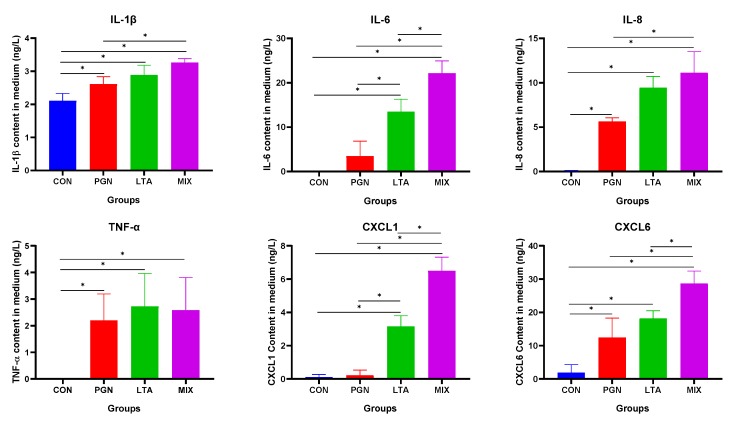
The proinflammatory factor concentrations in culture medium of different groups. The culture medium was collected to measure proinflammatory factor concentrations by enzyme-linked immunosorbent assay (ELISA). Data represent the mean and standard deviation (*n* = 6), and the asterisk indicates statistical difference (* *p* < 0.05) between the indicated columns, based on one-way analysis of variance and Duncan’s range test. IL-1β, interleukin-1β; IL-6, interleukin-6; IL-8, interleukin-8; TNF-α, tumor necrosis factor-α; CXCL1, chemokine (C-X-C motif) ligand 1; CXCL6, chemokine (C-X-C motif) ligand 6; CON, control group; PGN, peptidoglycan group; LTA, lipoteichoic acid group; MIX, PGN + LTA group.

The proinflammatory factor concentrations in culture medium of different groups were detected by enzyme-linked immunosorbent assay (ELISA). As shown in Figure 8, the concentrations of cytokines (IL-1β, IL-6, IL-8) and chemokines (CXCL1 and CXCL6) of the groups increased in the following order: CON < PGN < LTA < PGN + LTA group. The TNF-α concentration of the PGN, LTA, and PGN + LTA groups was also significantly higher than that of the CON group. 

#### 2.5.2. PGN and LTA Decreased Casein Synthesis 

**Figure 9:**
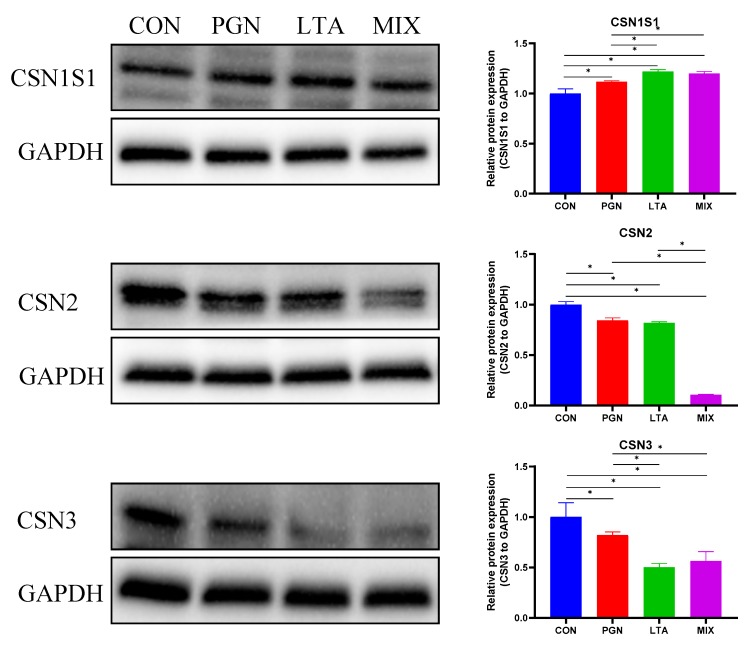
The protein expression of the three caseins (CSN1S1, CSN2, and CSN3). The total protein was isolated to examine casein expression by Western Blot analysis, and the glyceraldehyde-3-phosphate dehydrogenase (GAPDH) was selected as a housekeeping protein. Quantitation of blots is representative of three independent experiments. Data represent the mean and standard deviation (*n* = 6), and the asterisk indicates statistical difference (* *p* < 0.05) between the indicated columns, based on one-way analysis of variance and Duncan’s range test. CSN1S1, αS1-casein; CSN2, β-casein; CSN3, κ-casein; CON, control group; PGN, peptidoglycan group; LTA, lipoteichoic acid group; MIX, PGN + LTA group.

In order to evaluate the effects of PGN and LTA on milk protein synthesis in bovine mammary epithelial cells, the three caseins (CSN1S1, CSN2, and CSN3) were measured by Western Blot. As shown in Figure 9, the CSN1S1 expression of the PGN, LTA, and PGN + LTA groups was not suppressed, compared with the CON group. However, the protein expression of CSN2 and CSN3 for the PGN, LTA, and PGN + LTA groups was significantly lower than that of the CON group. 

## 3. Discussion

*S. aureus* is one of the most common gram-positive bacterial pathogens causing subclinical mastitis [23,24,25,26]. A lot of cell wall lysis components are released in the process of proliferation or/and death of *S. aureus* [27,28]. PGN and LTA are the major cell wall lysis components [29]. They can stimulate the bovine mammary gland to produce proinflammatory factors and then induce mastitis and reduce lactation [7,30,31,32]. However, their proinflammatory activity is weaker than that of LPS [2,3,4], a cell wall lysis component of gram-negative bacteria like *E. coli*. The weaker proinflammatory activity of PGN and LTA may explain why subclinical and chronic mastitis is mainly caused by gram-positive bacteria, while clinical and acute mastitis is mainly caused by gram-negative bacteria [3]. Therefore, mastitis caused by *S. aureus* is more difficult to detect and cure than that caused by most of the other bacterial pathogens [26,33]. Thus, it is important to study the effects of PGN and LTA from *S. aureus* on mastitis and lactation and the relevant mechanisms.

The RNA-Seq technology combined with bioinformatics analysis allows us to more rapidly and comprehensively explore the pathogenic role and pathways of a pathologic factor through probing gene expression profiling. The gene expression profiling after LTA (20 μg/mL) treatment of bovine mammary epithelial cells for 12 h had been characterized using RNA-Seq [5]. A total of 12 upregulated DEGs and 12 down-regulated DEGs had been found, and these majorities of DEGs were enriched in inflammation-related pathways [5]. The gene expression profiling after treatment of bovine mammary epithelial cells for 1 h with PGN (30 μg/mL) + LTA (30 μg/mL) from *S. aureus* had been characterized, and 14 inflammatory mediator-related DEGs and 17 inflammation-related DEGs had been found in a study [6]. Our present study further analyzed the gene expression profiling induced by PGN and LTA from *S. aureus* through RNA-Seq. The bovine mammary epithelial cells were not only stimulated by PGN or LTA alone, but also co-stimulated by PGN and LTA in our study. We found that the relative mRNA expression of proinflammatory factors of different groups increased in the following order: CON < PGN < LTA < PGN + LTA, and the proinflammatory factor concentrations in culture medium of different groups also increased in the above order. Other studies also have demonstrated the proinflammatory activity of PGN and LTA [34,35,36,37,38,39]. Furthermore, our results indicate that the proinflammatory activity of LTA was greater than that of PGN and they displayed an additive effect in proinflammatory activity. 

The proinflammatory factor expression induced by PGN and LTA might be regulated by epigenetic modifications. DNA methylation is a stable and irreversible epigenetic modification that silences gene expression and commonly occurs at cytosine residues of DNA [40]. In mammalian cells, a methylated group is added to the cytosine residue of DNA under the catalysis of DNMT to create 5-methylcytosine [41,42]. Thus, DNMT is the key enzyme in the process of DNA methylation [43]. Commensal microbes also have an ability to directly influence DNA methylation status of a host and therefore may alter disease development [44]. For example, microbiota colonization in the colon of neonatal mice resulted in hypomethylation of the *CXCL16* [45]. In addition to the direct effects of bacteria on DNA methylation, their cell wall lysis components, such as LPS, PGN, and LTA, also could regulate DNA methylation of a host. We had characterized the effects of different doses of LPS (0, 1, 10, 100, and 1000 endotoxin units/mL) on genome-wide DNA methylation of bovine mammary epithelial cells, using methylated DNA immunoprecipitation with high-throughput sequencing (MeDIP-seq) in our previous study [15]. LPS at higher doses induced hypomethylation of genes involved in the immune response pathway probably in favor of immune responses [15]. Although we could not find specific reports showing that LTA and PGN directly affect DNA methylation, LTA-deficient *Lactobacillus acidophilus* (NCK2025) could ameliorate inflammation-induced colitis-associated cancer by restoring aberrant DNA methylation status [44,46]. In addition, promoter hypomethylation of the Toll-like receptor-2 gene was associated with increased proinflammatory response toward PGN in cystic fibrosis bronchial epithelial cells [47]. In the present study, we found that LTA and LTA + PGN reduced DNA methylation by suppressing DNMT activity and increased proinflammatory factor expression. Hence, the results suggested that LTA or LTA + PGN resulted in hypomethylation of the genome and proinflammatory factor up-regulation. Although PGN also significantly promoted proinflammatory factor expression, the proinflammatory activity was weaker than that of LTA and its effect on DNA methylation and DNMT activity was not significant in our study, which might be due to the weak effect of PGN on DNMT activity and DNA methylation. 

In addition to DNA methylation, another classical epigenetic modification mechanism is histone acetylation, which is regulated by HAT and HDAC. As the names suggest, HAT and HDAC catalyze core histone acetylation and deacetylation at the N-terminal lysine residues, respectively, which enhance and suppress the DNA binding ability of histone, and then result in DNA condensation and de-condensation, respectively, leading to gene expression increase and decrease, respectively [42]. Hence, it is generally believed that a higher acetylation level of histone H3 or/and H4 promotes gene transcription [48,49]. LTA could bind closely to arginine-rich histone H3 and H4, which involves cell membrane disruption [50]. Moreover, the recruitment of drosophila histone deacetylase 1 (dHDAC1) might be impacted by PGN and LPS [51]. Therefore, we speculated that PGN and LTA might influence histone acetylation in bovine mammary epithelial cells. In this study, we found that PGN and LTA decreased histone H3 acetylation through suppressing HAT activity. In addition, the HDAC activity increased when bovine mammary epithelial cells were co-stimulated by PGN and LTA, which further reduced histone H3 acetylation. At the same time, compared with the CON group, the relative mRNA expression of *CSN1* and *CSN2* significantly decreased and the relative mRNA expression of *CSN3* also decreased numerically. Our previous study showed that LPS suppressed lactation-related gene expression due to reducing histones H3 acetylation by enhancing HDAC activity [16]. Taken together, our results revealed that PGN and LTA reduced histone H3 acetylation through regulating HAT and HDAC activity, which might result in lower mRNA expression of caseins. Meanwhile, the protein expression of CSN2 and CSN3 also was reduced, which was consistent with their gene expression. This indicated that casein expression decreased when inflammation occurred in the bovine mammary epithelial cells after being stimulated by the pathogenic factors [52,53,54]. 

## 4. Conclusions

In summary, PGN and LTA promoted the expression of proinflammatory factors probably due to inducing DNA hypomethylation by suppressing DNMT activity, whereas PGN and LTA decreased the gene expression of caseins possibly through reducing histone H3 acetylation by suppressing HAT or/and promoting HDAC activity. The influences of LTA on gene expression profiling and proinflammatory factor expression were greater than those of PGN and they displayed an additive effect on inflammation.

## 5. Materials and Methods

### 5.1. Cell Culture and Treatments

The bovine mammary epithelial cell line (MAC-T) was kindly provided by Professors Jianxin Liu and Hongyun Liu at the Institute for Dairy Research, Zhejiang University, China. The cell line has been widely used and its establishment method had been reported by Huynh et al. [55]. The cells were seeded into six-well plates (Corning, NY, USA) at a density of 1 × 10^5^ cells per well and cultured in complete culture medium, which contained 90% DMEM/F-12 (Gibco, Carlsbad, CA, USA), 10% FBS (Gibco, Carlsbad, CA, USA), 2.5 μg/mL insulin-transferrin-selenium (Gibco, Carlsbad, CA, USA), 1 μg/mL hydrocortisone (Solarbio, Beijing, China), 1 μg/mL progesterone (Solarbio, Beijing, China), and 5 ng/mL epidermal growth factor (Sigma-Aldrich, St. Louis, MO, USA). Each well of the six-well plates contained 2 mL culture medium, which was replaced per 24 h. The cells were incubated in an incubator (Thermo Fisher Scientific, Waltham, MA, USA) at 37 °C with 5% CO_2_.

The cells were treated for 24 h with neither PGN nor LTA (CON), PGN (30 μg/mL, Reference number: 77140-10MG; Sigma-Aldrich, St. Louis, MO, USA), LTA (30 μg/mL, Reference number: L2515-10MG; Sigma-Aldrich, St. Louis, MO, USA), and PGN + LTA (30 μg/mL for each). The dose of 30 μg/mL PGN or LTA can stimulate immunologically bovine mammary epithelial cells [6]. PGN and LTA were added into the culture medium when the cells grew to 60–70% confluence. Each treatment or group had six biological replicates (*n* = 6). The cells and the supernatant of culture medium were collected after 24 h for subsequent analyses. 

### 5.2. RNA-Seq Data Analysis 

Three cell samples for each group were used for RNA-Seq analysis. Total RNA extracting, library constructing, high-throughput sequencing, and bioinformatics analyzing were performed through custom service from Genedenovo Biotechnology Co., Ltd. (Guangzhou, China) (http://www.genedenovo.com/). Briefly, Total RNA was extracted using the Trizol reagent kit (Invitrogen, Carlsbad, CA, USA), and then was enriched by Oligo(dT). The sequencing was conducted using the Illumina HiSeq 2500 platform. The bioinformatics flow analysis was performed by the online OmicShare cloud platform (https://www.omicshare.com/tools/). 

### 5.3. Total RNA Isolation, Reverse Transcription, and RT-qPCR

Total RNAs were isolated from the collected cells using the TRIzol reagent (Invitrogen, Carlsbad, CA, USA) in accordance with the manufacturer’s protocols. The purity and concentration of RNA were measured by a spectrophotometer (Implen, Munich, Germany). Reverse transcription was performed using the iScript cDNA synthesis kit (Bio-Rad, Hercules, CA, USA) according to the manufacturer’s instructions for generating complementary DNA (cDNA). The incubation program of reverse transcription consisted of 25 °C for 5 min, 46 °C for 20 min, and 95 °C for 1 min. RT-qPCR reactions were performed in a BIO-RAD CFX96 Real-Time System (Bio-Rad, Hercules, CA, USA) using the Ssofast EvaGreen Supermix kit (Bio-Rad, Hercules, CA, USA) according to the manufacturer’s instructions. Every reaction system volume of 20 μL contained 800 ng cDNA. Each group had six biological replicates, and each cDNA sample was assayed three times. The cycling conditions for RT-qPCR were as follows: 95 °C for 30 sec, 40 cycles at 95 °C for 5 sec, and 58 °C for 5 sec. In order to ensure specific amplification, a melting curve analysis was conducted at the end of each RT-qPCR reaction program. The primer sequences (Table S11) were designed using NCBI primer-BLAST (https://www.ncbi.nlm.nih.gov/tools/primer-blast/) and synthesized by BGI Co., Ltd. (Shenzhen, China). The glyceraldehyde-3-phosphate dehydrogenase gene (GAPDH) was selected as a housekeeping gene for normalizing the expression data. The relative mRNA expression of target genes was calculated using the 2^−ΔΔCT^ method.

### 5.4. Analysis of Total DNA Methylation, Histone Acetylation, and Activity of DNMT, HAT and HDAC 

The total DNA of cell samples was isolated using the Ezup Column Animal Genomic DNA Purification Kit (Sangon Biotech, shanghai, China) for the DNA methylation analysis. The purity and concentration of DNA were measured by a spectrophotometer (Implen, Munich, Germany). The total DNA methylation levels were analyzed using the MethylFlash Global DNA Methylation (5-mC) ELISA Easy Kit (Epigentek Group Inc., Farmingdale, NY, USA). Nuclear protein was isolated using the Nucleoprotein Extraction Kit (Sangon Biotech, Shanghai, China) for histone acetylation and HAT and HDAC activity analyses. The concentration of nuclear protein was measured using the BCA Protein Assay Kit (Sangon Biotech, Shanghai, China) and a microplate reader (Thermo Fisher Scientific, Waltham, MA, USA). The acetylation levels of histone H3 and H4 were analyzed by Western Blot. The activity of DNMT, HAT, and HDAC was detected by the EpiQuik™ DNMT Activity/Inhibition Assay Ultra Kit (Epigentek Group Inc., Farmingdale, NY, USA), HAT Activity Colorimetric Assay Kit (BioVision, Milpitas, CA, USA), and HDAC Activity Colorimetric Assay Kit (BioVision, Milpitas, CA, USA), respectively. All assays were performed according to the manufacturer’s instructions. 

### 5.5. ELISA and Western Blot Assays

The proinflammatory factor concentrations in culture medium were detected by ELISA. The protein expression of caseins (CSN1, CSN2, and CSN3) and histones was analyzed by Western Blot. The six ELISA kits for measuring proinflammatory factors (IL-1β, IL-6, IL-8, TNF-α, CXCL1, and CXCL6) were purchased from Nanjing Jiancheng Bioengineering Institute (Nanjing, China). The total protein of cell samples was isolated using the Tissue or Cell Total Protein Extraction Kit (Sangon Biotech, Shanghai, China) for detecting casein expression. The extracting of nuclear protein and measuring of protein concentration had been described above. The 20 μg protein was separated using 4–20% SDS-PAGE (SurePAGETM, Bis-Tris, Genscript, Nanjing, China) at 140 V for 50 min and subsequently transferred to PVDF membranes (GE Healthcare, Wasukesha, WI, USA). The membranes were incubated overnight at 4 °C with primary antibody after blocking for 1 h with 5% skim milk buffer (BioFroxx, KG, Germany). The primary antibodies included CSN1S1 (Bioss Antibodies, bs-10034R, Beijing, China), CSN2 (Bioss Antibodies, bs-10032R, Beijing, China), CSN3 (Bioss Antibodies, bs-10031R, Beijing, China), Histone H3 (17168-1-AP, Proteintech, Wuhan, China), Histone H4 (16047-1-AP, Proteintech, Wuhan, China), acetylated Histone H3 (Acetyl Lys9/14, Bioss Antibodies, bs-4316R, Beijing, China), acetylated Histone H4 (Acetyl K5, Bioss Antibodies, bs-10721R, Beijing, China), and GAPDH (60004-1-Ig, Proteintech, Wuhan, China). The membranes were washed four times with TBST (APPLYGEN, Beijing, China) and incubated for 1 h at room temperature with secondary antibody, i.e., horseradish peroxidase (HRP)-conjugated Affinipure Goat Anti-Mouse IgG (H + L) (SA00001-1, Proteintech, Wuhan, China) or HRP-conjugated Affinipure Goat Anti-Rabbit IgG (H + L) (SA00001-2, Proteintech, Wuhan, China). Finally, the membranes were washed four times again with TBST and visualized using the ChemiDoc™ MP System (Bio-Rad, Hercules, CA, USA) with the Clarity Western ECL Substrate reagent (Bio-Rad, Hercules, CA, USA). The band density was analyzed using Image Lab 6.0.1 software (Bio-Rad, Hercules, CA, USA) and normalized by the GAPDH or Histone H3. 

### 5.6. Statistical Analysis

The data obtained are presented as means ± standard deviation (SD). Statistical analysis was performed using IBM SPSS statistics software 19.0 (IBM Inc., Armonk, NY, USA). One-way analysis of variance followed by Duncan’s multiple range test was used to determine statistical difference among the four groups. Probability values (*p*-values) less than 0.05 (*p* < 0.05) were considered as statistically significant. 

## Supplementary Materials

The following are available online at https://www.mdpi.com/2072-6651/12/4/238/s1, Table S1: The FastQC reports for RNA-Seq; Table S2: The annotations of all DEGs in the CON vs. PGN condition; Table S3: The annotations of all DEGs in the CON vs. LTA condition; Table S4: The annotations of all DEGs in the CON vs. PGN + LTA condition; Table S5: The top 10 GO biological process, molecular function, and cellular component terms of the DEGs of CON vs. PGN; Table S6: The top 10 GO biological process, molecular function, and cellular component terms of the DEGs of CON vs. LTA; Table S7: The top 10 GO biological process, molecular function, and cellular component terms of the DEGs of CON vs. MIX; Table S8: The annotations of all KEGG pathways in CON vs. PGN condition; Table S9: The annotations of all KEGG pathways in CON vs. LTA condition; Table S10: The annotations of all KEGG pathways in CON vs. PGN+LTA condition; Table S11: The parameters of primers for inflammation- and lactation-related genes and the GAPDH gene.

## References

[1] Szyda J., Mielczarek M., Fraszczak M., Minozzi G., Williams J.L., Wojdak-Maksymiec K. (2019). The genetic background of clinical mastitis in Holstein-Friesian cattle. Animal.

[2] Yu C., Shi Z.R., Chu C.Y., Lee K.H., Zhao X., Lee J.W. (2010). Expression of bovine granulocyte chemotactic protein-2 (GCP-2) in neutrophils and a mammary epithelial cell line (MAC-T) in response to various bacterial cell wall components. Vet. J..

[3] Strandberg Y., Gray C., Vuocolo T., Donaldson L., Broadway M., Tellam R. (2005). Lipopolysaccharide and lipoteichoic acid induce different innate immune responses in bovine mammary epithelial cells. Cytokine.

[4] Eckel E.F., Ametaj B.N. (2016). Invited review: Role of bacterial endotoxins in the etiopathogenesis of periparturient diseases of transition dairy cows. J. Dairy Sci..

[5] Xu T., Deng R., Li X., Zhang Y., Gao M. (2019). RNA-seq analysis of different inflammatory reactions induced by lipopolysaccharide and lipoteichoic acid in bovine mammary epithelial cells. Microb. Pathog..

[6] Im J., Lee T., Jeon J.H., Baik J.E., Kim K.W., Kang S.-S., Yun C.-H., Kim H., Han S.H. (2014). Gene expression profiling of bovine mammary gland epithelial cells stimulated with lipoteichoic acid plus peptidoglycan from *Staphylococcus aureus*. Int. Immunopharmacol..

[7] Kiku Y., Nagasawa Y., Tanabe F., Sugawara K., Watanabe A., Hata E., Ozawa T., Nakajima K., Arai T., Hayashi T. (2016). The cell wall component lipoteichoic acid of *Staphylococcus aureus* induces chemokine gene expression in bovine mammary epithelial cells. J. Vet. Med. Sci..

[8] Sun P., Zhang S.J., Maksim S., Yao Y.F., Liu H.M., Du J. (2019). Epigenetic modification in macrophages: A promising target for tumor and inflammation-associated disease therapy. Curr. Top. Med. Chem..

[9] Zong D.D., Ouyang R.Y., Chen P. (2015). Epigenetic mechanisms in chronic obstructive pulmonary disease. Eur. Rev. Med. Pharmacol. Sci..

[10] Holliday R., Pugh J.E. (1975). DNA modification mechanisms and gene activity during development. Science.

[11] Sajjanar B., Trakooljul N., Wimmers K., Ponsuksili S. (2019). DNA methylation analysis of porcine mammary epithelial cells reveals differentially methylated loci associated with immune response against *Escherichia coli* challenge. BMC Genom..

[12] Mo Z., Li Q., Cai L., Zhan M., Xu Q. (2019). The effect of DNA methylation on the miRNA expression pattern in lipopolysaccharide-induced inflammatory responses in human dental pulp cells. Mol. Immunol..

[13] Feng Z., Zhan M., Meng R., Wang X., Xu Q. (2019). 5-Aza-2’-deoxycytidine enhances lipopolysaccharide-induced inflammatory cytokine expression in human dental pulp cells by regulating TRAF6 methylation. Bioengineered.

[14] Korkmaz F.T., Kerr D.E. (2017). Genome-wide methylation analysis reveals differentially methylated loci that are associated with an age-dependent increase in bovine fibroblast response to LPS. BMC Genom..

[15] Chen J., Wu Y., Sun Y., Dong X., Wang Z., Zhang Z., Xiao Y., Dong G. (2019). Bacterial lipopolysaccharide induced alterations of genome-wide DNA methylation and promoter methylation of lactation-related genes in bovine mammary epithelial cells. Toxins.

[16] Chen J., Wu Y., Sun Y., Dong X., Wang Z., Zhang Z., Xiao Y., Dong G. (2019). Bacterial endotoxin decreased histone H3 acetylation of bovine mammary epithelial cells and the adverse effect was suppressed by sodium butyrate. BMC Vet. Res..

[17] Song M., He Y., Zhou H., Zhang Y., Li X., Yu Y. (2016). Combined analysis of DNA methylome and transcriptome reveal novel candidate genes with susceptibility to bovine *Staphylococcus aureus* subclinical mastitis. Sci. Rep..

[18] Zhang Y., Wang X., Jiang Q., Hao H., Ju Z., Yang C., Sun Y., Wang C., Zhong J., Huang J. (2018). DNA methylation rather than single nucleotide polymorphisms regulates the production of an aberrant splice variant of IL6R in mastitic cows. Cell Stress Chaperones.

[19] Wang D., Wei Y., Shi L., Khan M.Z., Fan L., Wang Y., Yu Y. (2019). Genome-wide DNA methylation pattern in a mouse model reveals two novel genes associated with *Staphylococcus aureus* mastitis. Asian-Australas. J. Anim. Sci..

[20] Tesfaye G.Y., Regassa F.G., Kelay B. (2010). Milk yield and associated economic losses in quarters with subclinical mastitis due to *Staphylococcus aureus* in Ethiopian crossbred dairy cows. Trop. Anim. Health Prod..

[21] Choudhury A., Solanki B., Singh S., Sahu U., Parvez S., Kar S., Ganguly S. (2019). Persistent peripheral presence of *Staphylococcus aureus* promotes histone H3 hypoacetylation and decreases tyrosine hydroxylase protein level in rat brain tissues. Neuroreport.

[22] Zhang Q.L., Su L.D., Lu Z.Q. (2015). Expression of DNA methylation and histone acetylation related genes in response to bacterial infection in the silkworm, Bombyx mori. Acta Entomol. Sin..

[23] Barkema H.W., Schukken Y.H., Lam T., Beiboer M.L., Wilmink H., Benedictus G., Brand A. (1998). Incidence of clinical mastitis in dairy herds grouped in three categories by bulk milk somatic cell counts. J. Dairy Sci..

[24] Riollet C., Rainard P., Poutrel B. (2001). Cell subpopulations and cytokine expression in cow milk in response to chronic *Staphylococcus aureus* infection. J. Dairy Sci..

[25] Barkema H.W., Schukken Y.H., Zadoks R.N. (2006). Invited review: The role of cow, pathogen, and treatment regimen in the therapeutic success of bovine *Staphylococcus aureus* mastitis. J. Dairy Sci..

[26] Zaatout N., Ayachi A., Kecha M., Kadlec K. (2019). Identification of staphylococci causing mastitis in dairy cattle from Algeria and characterization of *Staphylococcus aureus*. J. Appl. Microbiol..

[27] Pidgeon S.E., Pires M.M. (2017). Cell wall remodeling of *staphylococcus aureus* in live caenorhabditis elegans. Bioconj. Chem..

[28] Steven B., Maria J.P.S., Walker S. (2013). Wall teichoic acids of gram-positive bacteria. Annu. Rev. Microbiol..

[29] Rajagopal M., Walker S., Bagnoli F., Rappuoli R. (2017). Envelope structures of Gram-positive bacteria. Protein and Sugar Export and Assembly in Gram-Positive Bacteria.

[30] Kratochvilova L., Kharkevich K., Slama P. (2018). TNF-alpha and IL-10 are Produced by Leukocytes During the Experimental Inflammatory Response of Bovine Mammary Gland Induced by Peptidoglycan.

[31] Kharkevich K., Kratochvilova L., Slama P. (2018). Transforming Growth Factor Beta 1 Production During Inflammatory Response of Mammary Gland Induced by Peptidoglycan.

[32] Zhang W.Y., Li X.Z., Xu T., Ma M.R., Zhang Y., Gao M.Q. (2016). Inflammatory responses of stromal fibroblasts to inflammatory epithelial cells are involved in the pathogenesis of bovine mastitis. Exp. Cell Res..

[33] Sartori C., Boss R., Bodmer M., Leuenberger A., Ivanovic I., Graber H.U. (2018). Sanitation of *Staphylococcus aureus* genotype B-positive dairy herds: A field study. J. Dairy Sci..

[34] Vangan N., Cao Y.F., Jia X.Y., Bao W.L., Wang Y.F., He Q., Binderiya U., Feng X., Li T.T., Hao H.F. (2016). mTORC1 mediates peptidoglycan induced inflammatory cytokines expression and NF-kappa B activation in macrophages. Microb. Pathog..

[35] Zingarelli B. (2004). Peptidoglycan is an important pathogenic factor of the inflammatory response in sepsis. Crit. Care Med..

[36] Iyer J.K., Khurana T., Langer M., West C.M., Ballard J.D., Metcalf J.P., Merkel T.J., Coggeshall K.M. (2010). Inflammatory cytokine response to bacillus anthracis peptidoglycan requires phagocytosis and lysosomal trafficking. Infect. Immun..

[37] Boveri M., Kinsner A., Berezowski V., Lenfant A.M., Draing C., Cecchelli R., Dehouck M.P., Hartung T., Prieto P., Bal-Price A. (2006). Highly purified lipoteichoic acid from gram-positive bacteria induces In Vitro blood-brain barrier disruption through glia activation: Role of pro-inflammatory cytokines and nitric oxide. Neuroscience.

[38] Wang J.E., Dahle M.K., McDonald M., Foster S.J., Aasen A.O., Thiemermann C. (2003). Peptidoglycan and lipoteichoic acid in gram-positive bacterial sepsis: Receptors, signal transduction, biological effects, and synergism. Shock.

[39] Kang S.S., Sim J.R., Yun C.H., Han S.H. (2016). Lipoteichoic acids as a major virulence factor causing inflammatory responses via Toll-like receptor 2. Arch. Pharmacal Res..

[40] Hermann A., Gowher H., Jeltsch A. (2004). Biochemistry and biology of mammalian DNA methyltransferases. Cell. Mol. Life Sci..

[41] Chen T.P., Li E., Schatten G.P. (2004). Structure and function of eukaryotic DNA methyltransferases. Stem Cells in Development and Disease.

[42] Yen C., Huang H., Shu C., Hou M., Yuan S.F., Wang H., Chang Y., Farooqi A.A., Tang J., Chang H. (2016). DNA methylation, histone acetylation and methylation of epigenetic modifications as a therapeutic approach for cancers. Cancer Lett..

[43] Xu F., Mao C., Ding Y., Rui C., Wu L., Shi A., Zhang H., Zhang L., Xu Z. (2010). Molecular and enzymatic profiles of mammalian DNA methyltransferases: Structures and targets for drugs. Curr. Med. Chem..

[44] Low D., Mizoguchi A., Mizoguchi E. (2013). DNA methylation in inflammatory bowel disease and beyond. World J. Gastroenterol..

[45] Olszak T., An D., Zeissig S., Vera M.P., Richter J., Franke A., Glickman J.N., Siebert R., Baron R.M., Kasper D.L. (2012). Microbial exposure during early life has persistent effects on natural killer t cell function. Science.

[46] Lightfoot Y.L., Yang T., Sahay B., Mohamadzadeh M. (2013). Targeting aberrant colon cancer-specific DNA methylation with lipoteichoic acid-deficient Lactobacillus acidophilus. Gut Microbes.

[47] Shuto T., Furuta T., Oba M., Xu H.D., Li J.D., Cheung J., Gruenert D.C., Uehara A., Suico M.A., Okiyoneda T. (2006). Promoter hypomethylation of Toll-like receptor-2 gene is associated with increased proinflammatory response toward bacterial peptidoglycan in cystic fibrosis bronchial epithelial cells. FASEB J..

[48] Heintzman N.D., Hon G.C., Hawkins R.D., Kheradpour P., Stark A., Harp L.F., Ye Z., Lee L.K., Stuart R.K., Ching C.W. (2009). Histone modifications at human enhancers reflect global cell-type-specific gene expression. Nature.

[49] Deckert J., Struhl K. (2001). Histone acetylation at promoters is differentially affected by specific activators and repressors. Mol. Cell. Biol..

[50] Morita S., Tagai C., Shiraishi T., Miyaji K., Iwamuro S. (2013). Differential mode of antimicrobial actions of arginine-rich and lysine-rich histones against Gram-positive *Staphylococcus aureus*. Peptides.

[51] Kim T., Yoon J., Cho H.S., Lee W.B., Kim J., Song Y.H., Kim S.N., Yoon J.H., Kim-Ha J., Kim Y.J. (2005). Downregulation of lipopolysaccharide response in drosophila by negative crosstalk between the AP1 and NF-kappa B signaling modules. Nat. Immunol..

[52] Cheng W.N., Jeong C.H., Seo H.G., Han S.G. (2019). Moringa extract attenuates inflammatory responses and increases gene expression of casein in bovine mammary epithelial cells. Animals.

[53] Wu T., Wang C., Ding L., Shen Y., Cui H., Wang M., Wang H. (2016). Arginine relieves the inflammatory response and enhances the casein expression in bovine mammary epithelial cells induced by lipopolysaccharide. Mediators Inflamm..

[54] Zhang K., Chang G., Xu T., Xu L., Guo J., Jin D., Shen X. (2016). Lipopolysaccharide derived from the digestive tract activates inflammatory gene expression and inhibits casein synthesis in the mammary glands of lactating dairy cows. Oncotarget.

[55] Huynh H.T., Robitaille G., Turner J.D. (1991). Establishment of bovine mammary epithelial-cells (mac-t)—An In Vitro model for bovine lactation. Exp. Cell Res..

