# Supplementary material for: PGN and LTA from Staphylococcus aureus Induced Inflammation and Decreased Lactation through Regulating DNA Methylation and Histone H3 Acetylation in Bovine Mammary Epithelial Cells
Source: Toxins (Basel). 2020 Apr 9;12(4):238. doi: 10.3390/toxins12040238 (PMC7232188; doi:10.3390/toxins12040238)
Supplement: Supplementary file 1 [file toxins-12-00238-s001.zip › toxins-732036-for conversion/Table S11. The parameters of primers.docx]

**Table S11.** The parameters of primers for inflammation- and lactation-related genes and the GAPDH gene.

| **Genes** | **Primer sequences (5' to 3')** | **Product Size/bp** | **Accession Number** |
| --- | --- | --- | --- |
| *IL-1β* | F: AGTGCCTACGCACATGTCTTC  R: TGCGTCACACAGAAACTCGTC | 114 | [NM_174093.1](https://www.ncbi.nlm.nih.gov/entrez/viewer.fcgi?db=nucleotide&id=27806570) |
| *IL-6* | F: TGCTGGTCTTCTGGAGTATC  R: GTGGCTGGAGTGGTTATTAG | 153 | [NM_173923.2](https://www.ncbi.nlm.nih.gov/entrez/viewer.fcgi?db=nucleotide&id=31343255) |
| *IL-8* | F: ATGACTTCCAAGCTGGCTGTTG  R: TTGATAAATTTGGGGTGGAAAG | 149 | [NM_173925.2](https://www.ncbi.nlm.nih.gov/entrez/viewer.fcgi?db=nucleotide&id=31343250) |
| *CXCL1* | F: ACCTCAAGAACATCCAGAGCG  R: GCTGGAGTATCAAGAAGCTCGT | 203 | [NM_175700.2](https://www.ncbi.nlm.nih.gov/entrez/viewer.fcgi?db=nucleotide&id=1199701052) |
| *CXCL6* | F: CCTCTGCAGTCCTCTCTTCG  R: TGGGATGAATTCCCGGTGTG | 203 | [NM_174300.2](https://www.ncbi.nlm.nih.gov/entrez/viewer.fcgi?db=nucleotide&id=31342385) |
| *TNF-α* | F: CCACGTTGTAGCCGACATC  R: CCCTGAAGAGGACCTGTGAG | 155 | [XM_005223596.4](https://www.ncbi.nlm.nih.gov/entrez/viewer.fcgi?db=nucleotide&id=1387237779) |
| *CSN1S1* | F: CTTTTCAGACAATTCTACCAGCT  R: AATTCACTTGACTCCTCACCAC | 171 | [NM_181029.2](https://www.ncbi.nlm.nih.gov/entrez/viewer.fcgi?db=nucleotide&id=31341348) |
| *CSN2* | F: AGTCCAAAGTCCTGCCTGTTCC  R: TGCCATATTTCCAGTCGCAGTC | 193 | [XM_015471671.2](https://www.ncbi.nlm.nih.gov/entrez/viewer.fcgi?db=nucleotide&id=1387273718) |
| *CSN3* | F: CAATACGCTGTGAGAAAGATGA  R: AACTGGTTTCTGTTGGTAGTAA | 122 | [NM_174294.2](https://www.ncbi.nlm.nih.gov/entrez/viewer.fcgi?db=nucleotide&id=402693386) |
| *GAPDH* | F: GGGTCATCATCTCTGCACCT  R: GGTCATAAGTCCCTCCACGA | 176 | [NM_001034034.2](https://www.ncbi.nlm.nih.gov/entrez/viewer.fcgi?db=nucleotide&id=402744670) |

*IL-1β*, interleukin-1β; *IL-6*, interleukin-6; *IL-8*, interleukin-8; *CXCL1*, chemokine (C-X-C motif) ligand 1; *CXCL6*, chemokine (C-X-C motif) ligand 6; *TNF-α*, tumor necrosis factor-α; *CSN1S1*, αS1-casein; *CSN2*, β-casein; *CSN3*, κ-casein.
