# Supplementary material for: PGN and LTA from Staphylococcus aureus Induced Inflammation and Decreased Lactation through Regulating DNA Methylation and Histone H3 Acetylation in Bovine Mammary Epithelial Cells
Source: Toxins (Basel). 2020 Apr 9;12(4):238. doi: 10.3390/toxins12040238 (PMC7232188; doi:10.3390/toxins12040238)
Supplement: Supplementary file 1 [file toxins-12-00238-s001.zip › toxins-732036-for conversion/Table S5. The top 10 GO biological process, molecular function, and cellular component terms of the differentially expressed genes (DEGs) of CON-vs-PGN..docx]

**Table S5.** The top 10 GO biological process, molecular function, and cellular component terms of the differentially expressed genes (DEGs) of CON-vs-PGN.

| **GO ID** | **Description** | ***p*-value** |
| --- | --- | --- |
| **Molecular Function** | | |
| GO:0005125 | cytokine activity | 0.000524 |
| GO:0042887 | amide transmembrane transporter activity | 0.003973 |
| GO:0005102 | receptor binding | 0.006286 |
| GO:0005539 | glycosaminoglycan binding | 0.009664 |
| GO:0019201 | nucleotide kinase activity | 0.011874 |
| GO:0045236 | CXCR chemokine receptor binding | 0.027491 |
| GO:0051393 | alpha-actinin binding | 0.027491 |
| GO:0017136 | NAD-dependent histone deacetylase activity | 0.031357 |
| GO:0034979 | NAD-dependent protein deacetylase activity | 0.031357 |
| GO:0016647 | oxidoreductase activity, acting on the CH-NH group of donors, oxygen as acceptor | 0.033116 |
| **Biological Process** | | |
| GO:0002376 | immune system process | 0.000602 |
| GO:0006952 | defense response | 0.000939 |
| GO:0014821 | phasic smooth muscle contraction | 0.001479 |
| GO:0002274 | myeloid leukocyte activation | 0.002185 |
| GO:0007155 | cell adhesion | 0.003403 |
| GO:0001775 | cell activation | 0.003756 |
| GO:0006598 | polyamine catabolic process | 0.004041 |
| GO:0022610 | biological adhesion | 0.004232 |
| GO:0003330 | regulation of extracellular matrix constituent secretion | 0.004463 |
| GO:0009310 | amine catabolic process | 0.004704 |
| **Cell Component** | | |
| GO:0031672 | A band | 0.001049 |
| GO:0044421 | extracellular region part | 0.006854 |
| GO:0097232 | lamellar body membrane | 0.008143 |
| GO:0005576 | extracellular region | 0.008214 |
| GO:0030017 | sarcomere | 0.008271 |
| GO:0044449 | contractile fiber part | 0.008495 |
| GO:0098802 | plasma membrane receptor complex | 0.012089 |
| GO:0030016 | myofibril | 0.014339 |
| GO:0008305 | integrin complex | 0.016221 |
| GO:0098636 | protein complex involved in cell adhesion | 0.016221 |
