# Supplementary material for: PGN and LTA from Staphylococcus aureus Induced Inflammation and Decreased Lactation through Regulating DNA Methylation and Histone H3 Acetylation in Bovine Mammary Epithelial Cells
Source: Toxins (Basel). 2020 Apr 9;12(4):238. doi: 10.3390/toxins12040238 (PMC7232188; doi:10.3390/toxins12040238)
Supplement: Supplementary file 1 [file toxins-12-00238-s001.zip › toxins-732036-for conversion/Table S6. The top 10 GO biological process, molecular function, and cellular component terms of the DEGs of CON-vs-LTA.docx]

**Table S6.** The top 10 GO biological process, molecular function, and cellular component terms of the DEGs of CON-vs-LTA.

| **GO ID** | **Description** | ***p*-value** |
| --- | --- | --- |
| **molecular function** | | |
| GO:0005125 | cytokine activity | 1.35E-05 |
| GO:0016709 | oxidoreductase activity, acting on paired donors, with incorporation or reduction of molecular oxygen, NAD(P)H as one donor, and incorporation of one atom of oxygen | 2.48E-05 |
| GO:0016705 | oxidoreductase activity, acting on paired donors, with incorporation or reduction of molecular oxygen | 0.000601 |
| GO:0016491 | oxidoreductase activity | 0.001133 |
| GO:0004497 | monooxygenase activity | 0.001525 |
| GO:0016647 | oxidoreductase activity, acting on the CH-NH group of donors, oxygen as acceptor | 0.002355 |
| GO:0016645 | oxidoreductase activity, acting on the CH-NH group of donors | 0.004294 |
| GO:0042927 | siderophore transporter activity | 0.007777 |
| GO:0070643 | vitamin D 25-hydroxylase activity | 0.007777 |
| GO:0005539 | glycosaminoglycan binding | 0.010905 |
| **biological process** | | |
| GO:0008203 | cholesterol metabolic process | 9.84E-07 |
| GO:1902652 | secondary alcohol metabolic process | 9.84E-07 |
| GO:0016125 | sterol metabolic process | 3.54E-06 |
| GO:0006066 | alcohol metabolic process | 1.00E-05 |
| GO:0008202 | steroid metabolic process | 4.22E-05 |
| GO:0006629 | lipid metabolic process | 5.82E-05 |
| GO:1901615 | organic hydroxy compound metabolic process | 9.20E-05 |
| GO:0032963 | collagen metabolic process | 0.000174 |
| GO:0001818 | negative regulation of cytokine production | 0.000222 |
| GO:0044255 | cellular lipid metabolic process | 0.000238 |
| **cellular component** | | |
| GO:0042175 | nuclear outer membrane-endoplasmic reticulum membrane network | 0.002711 |
| GO:0048180 | activin complex | 0.007519 |
| GO:0043511 | inhibin complex | 0.014983 |
| GO:0097232 | lamellar body membrane | 0.014983 |
| GO:0005615 | extracellular space | 0.043148 |
| GO:0000808 | origin recognition complex | 0.058612 |
| GO:0042599 | lamellar body | 0.065695 |
| GO:0031012 | extracellular matrix | 0.066729 |
| GO:0005882 | intermediate filament | 0.09267 |
| GO:0030176 | integral component of endoplasmic reticulum membrane | 0.093504 |
