# Supplementary material for: PGN and LTA from Staphylococcus aureus Induced Inflammation and Decreased Lactation through Regulating DNA Methylation and Histone H3 Acetylation in Bovine Mammary Epithelial Cells
Source: Toxins (Basel). 2020 Apr 9;12(4):238. doi: 10.3390/toxins12040238 (PMC7232188; doi:10.3390/toxins12040238)
Supplement: Supplementary file 1 [file toxins-12-00238-s001.zip › toxins-732036-for conversion/Table S7. The top 10 GO biological process, molecular function, and cellular component terms of the DEGs of CON-vs-MIX.docx]

**Table S7.** The top 10 GO biological process, molecular function, and cellular component terms of the DEGs of CON-vs-MIX.

| **GO ID** | **Description** | ***p*-value** |
| --- | --- | --- |
| **molecular function** | | |
| GO:0097367 | carbohydrate derivative binding | 1.70E-06 |
| GO:0001883 | purine nucleoside binding | 4.03E-06 |
| GO:0032549 | ribonucleoside binding | 4.03E-06 |
| GO:0032550 | purine ribonucleoside binding | 4.03E-06 |
| GO:0001882 | nucleoside binding | 4.20E-06 |
| GO:0003774 | motor activity | 1.12E-05 |
| GO:0016772 | transferase activity, transferring phosphorus-containing groups | 2.58E-05 |
| GO:0036094 | small molecule binding | 3.69E-05 |
| GO:0016773 | phosphotransferase activity, alcohol group as acceptor | 9.58E-05 |
| GO:0005515 | protein binding | 0.000139 |
| **biological process** | | |
| GO:0000278 | mitotic cell cycle | 6.14E-10 |
| GO:1903047 | mitotic cell cycle process | 1.15E-09 |
| GO:0007059 | chromosome segregation | 1.20E-09 |
| GO:0022402 | cell cycle process | 3.78E-09 |
| GO:0007049 | cell cycle | 5.97E-09 |
| GO:0098813 | nuclear chromosome segregation | 3.20E-08 |
| GO:0000819 | sister chromatid segregation | 4.02E-08 |
| GO:0000280 | nuclear division | 7.69E-08 |
| GO:0007067 | mitotic nuclear division | 9.33E-08 |
| GO:0048285 | organelle fission | 1.89E-07 |
| **cellular component** | | |
| GO:0044430 | cytoskeletal part | 7.27E-11 |
| GO:0015630 | microtubule cytoskeleton | 7.67E-10 |
| GO:0005694 | chromosome | 7.75E-10 |
| GO:0043228 | non-membrane-bounded organelle | 1.57E-09 |
| GO:0043232 | intracellular non-membrane-bounded organelle | 1.57E-09 |
| GO:0005819 | spindle | 1.65E-08 |
| GO:0000793 | condensed chromosome | 2.78E-08 |
| GO:0005856 | cytoskeleton | 5.28E-08 |
| GO:0044427 | chromosomal part | 6.09E-08 |
| GO:0000775 | chromosome, centromeric region | 1.85E-07 |
